# Circular RNA mediated gene regulation in chronic diabetic complications

**DOI:** 10.1038/s41598-021-02980-y

**Published:** 2021-12-09

**Authors:** Nikhil S. Patil, Biao Feng, Zhaoliang Su, Christina A. Castellani, Subrata Chakrabarti

**Affiliations:** 1 Michael G Degroote School of Medicine, McMaster University, Hamilton, ON Canada; 2 Department of Pathology and Laboratory Medicine, Western University, London, ON N6A 5C1 Canada; 3 Department of Immunology, Jiangsu University, Jiangsu, People’s Republic of China

**Keywords:** Epigenetics, Genetics research, Diabetes

## Abstract

Chronic diabetic complications affect multiple organs causing widespread organ damage. Although there are some commonalities, the phenotype of such changes show tissue specific variation. Given this, we examined whether differences in circular RNA (circRNA) mediated gene regulatory mechanisms contribute to changes in gene expression at the basal level and in diabetes. CircRNAs are single-stranded RNA with covalently closed loop structures and act as miRNA sponges, factors of RNA splicing, scaffolding for proteins, regulators of transcription, and modulators of the expression of parental genes, among other roles. We examined heart and retinal tissue from Streptozotocin-induced diabetic mice with established diabetes related tissue damage and tissue from non-diabetic controls. A custom array analysis was performed and the data were analysed. Two major circRNA mediated processes were uniquely upregulated in diabetic heart tissue, namely, positive regulation of endothelial cell migration and regulation of mitochondria: mitochondrial electron transport. In the retina, circRNAs regulating extracellular matrix protein production and endothelial to mesenchymal transition (EndMT) were found to be upregulated. The current study identified regulatory and potential pathogenetic roles of specific circRNA in diabetic retinopathy and cardiomyopathy. Understanding such novel mechanisms, may in the future, be useful to develop RNA based treatment strategies.

## Introduction

In diabetes, hyperglycemia causes changes in cellular transcription. Endothelial cells (ECs) alter their synthetic phenotype when in a high glucose environment, and subsequently affect other cells in the target organs of diabetic complications including the heart and retina^1,2^. Glucose-induced biochemical alterations converge on the EC nucleus and change gene transcription^3,4^.

Alterations of inflammatory cytokines, extracellular matrix (ECM) proteins and aberrant angiogenesis are some of the key pathologic processes that cause impaired cellular and organ functions^1–4^. Although some of the pathologic processes are similar in the retina and heart, such as increased ECM protein production, others, such as angiogenesis, vary between these organs^5,6^. Angiogenesis is a main feature in proliferative diabetic retinopathy, however this process becomes impaired in the diabetic heart^7^. Hence, transcriptional and post-transcriptional regulatory mechanisms likely vary between different diabetic organs.. Previously, we have identified that a concerted effort of multiple transcription factors, transcription co-activators and microRNAs/long non-coding RNAs regulate specific vasoactive molecules and ECM proteins in the context of diabetic complications^8–11^. However, other recently identified non-coding RNAs such as circular RNAs (circRNAs) also play a significant role in this concert.

CircRNAs are single-stranded RNA molecules. In contrast to linear RNAs, they demonstrate closed loop structures generated as a result of back-splicing^12^. Due to circularisation, these molecules are devoid of 5-prime caps or poly-A tails. Originally discovered more than fourdecades ago, in recent yearsnew sequencing technologies have been developed allowing for the identificationof circular RNA isoforms from a large number of genes, and demonstratingthat these circRNAs are transcribed in both humans and mice^13,14^. In addition, expression of circRNAS is cell type dependent. It has been estimated that in human, circRNA expression may account for 1% of mRNAs^14^. The most common mechanism of circularization involves the spliceosome machinery and occurs in pre-messenger RNAs conventionally transcribed by RNA polymerase II (RNAP II) from nuclear-encoded genes. Physiological RNA circularization by the spliceosome can proceed by three principle mechanisms^15,16^. CircRNAs exhibit higher stability and act as miRNA sponges, factors of RNA splicing, scaffolding for proteins, regulators of transcription, and modulators of the expression of parental genes. circRNAs can also serve as biomarkers for numerous diseases^17–19^. To date, several circRNAs have been functionally studied in the context of cardiometabolic disease^20^, The ubiquity of circRNA and their specific regulation could significantly alter our perspective on post-transcriptional regulation and the roles that RNA can play in the cell, making circRNAs a promising candidate for diagnostic modalities and therapies.

Overall, non-coding RNAs play a significant role in all biological processes and diseases. The role of circRNAs has become increasingly important among non-coding RNAs. The availability of RNA deep sequencing and bioinformatics has started to reveal the importance of circRNAs as regulators of gene expression in chronic diabetic compliations. Specifically, in the retina, circHIPK3 acts as an endogenous miR-30a-3p sponge to sequester and inhibit miR-30a-3p activity, which leads to increased vascular endothelial growth factor-C expression^21^. Studies have demonstrated that circRNAs can be methylated by N6-methyladenosine (m6A), which is the most abundant base modification of RNA, leading to promotion of efficient initiation of protein translation from circRNAs in human cells^16,22^. m6A translation is enhanced by METTL3 (methyltransferase-like 3) and METTL14 (methyltransferase-like 14), and inhibited by demethylase FTO (obesity-associated protein)^22^. CircRNA Col1A2 was also found to promote angiogenesis through miR29b/VEGF^23^. Other circular RNAs, altered in diabetic retinopathy include circular DNMT3B and circRNA_0084043, each working through various pathways^24,25^. In the heart, 58 significantly differentially expressed circRNAs were identified in db/db mice, a model of type 2 diabetes^26^. Also identified are alterations of circRNA_010567 and circRNA_000203 working through various pathways to regulate specific transcript altering fibrosis related genes^27,28^.

The identification of differential expression of various circular RNAs in the heart and retina is important to understanding disease etiologyas diabetes affects these organs differently. For example, abnormal angiogenesis is seen in the retina in diabetes, whereas lack of angiogenesis is observed in the heart^6,7^. Hence, it is conceptually possible that in diabetes, gene transcription and the regulatory mechanisms thereof, will also be different in these two organs. The aim of the current study if to have a better understanding of the differential expression of circRNA expression in both the basal and diabetic state. This approach will lead to better understanding of the pathogenetic mechanisms of transcription and subsequent tissue damage in diabetic patients.

## Methods

### Animal models

The Western University Council for Animal Care Committee approved all animal experiments, which were performed in accordance with *The Guide for the Care and Use of Laboratory Animals* (NIH Publication 85–23, revised in 1996). Western’s Animal Care Committee is responsible for overseeing all aspects of animal ethics, care and use. Mice (C57/BL6 background; 22–24 g, 8 weeks old) were obtained (Charles River, Wilmington, USA) and randomly divided into control and diabetic groups. As our previous data were obtained from male mice and for cost containment, we used only male mice for this initial study. Streptozotocin (STZ) (50 mg/kg IP, 5 injection on consecutive days) was used to generate a type 1 diabetic animal model. Age- and sex-matched littermate controls received identical volumes of citrate buffer. Diabetes was confirmed by measuring blood glucose (> 16.7 mmol/L) from a tail vein using a glucometer. Animals were monitored for changes in body weight and blood glucose. After 8 weeks of diabetes, mice (n = 6/group) were euthanized. Retinal and left ventricular tissues were collected and immediately frozen for further analysis. A small portion of the cardiac tissue from each mouse was formalin fixed, paraffin embedded and stained with hematoxylin/eosin and trichome stain for morphologic analysis. Animal monitoring and tissue collection have been previously described^29,30^. The microarray study (please see below) included 3 control mice and 3 diabetic mice, with both retina and heart samples collected from each of the 6 mice. This animal study is reported in accordance with ARRIVE guidelines.

### Echocardiography

Echocardiography was used to measure possible cardiac functional alterations in diabetes using previously described methodology^29,30^. Animals were anesthetized (1.5% inhaled isoflurane) and examined on a warm handling platform. A 40-MHz linear array transducer (MS-550D) and Vevo 2100 preclinical ultrasound system (VisualSonics) was used. Left ventricular fractional shortening (FS) was used as the cardiac contractile function index. Pulse-waved color flow-guided Doppler recordings of maximal early (E) and late (A) diastolic transmittal flow velocities and Doppler tissue imaging recordings of peak E = velocity and peak A = velocity were collected. Mitral inflow patterns (E/A ratio) was used to assess diastolic dysfunction as described^29,30^.

### Histological analysis

Tissues collected in formalin were embedded in paraffin and 5 µm sections were cut. The tissues were stained with hematoxylin and eosin and trichrome stain following standard procedure^29,30^.

### RNA analysis

TRIzol™(Invitrogen) was used to extract total RNA. The quality of the extracted RNA was checked spectrophotometrically and via gel analyses. From a portion of the extracted RNA, cDNA for PCR was synthesized using high-capacity cDNA reverse-transcription kit (Applied Biosystems, Burlington, ON). To examine transcriptional alterations in diabetes, mRNA expression of specific transcripts (Collagen, fibronectin) were performed using real-time RT-PCR using a LightCycler (Roche Diagnostics). The housekeeping gene β-actin was used to normalize the data^10,29,30^.

The remaining RNA samples were then shipped to Arraystar for circular RNA array analysis using the Arraystar Mouse circRNA Array V2 (8 × 15 K) panel.

### circRNA microarray

The purity and concentration of total RNA from each sample was quantified using the NanoDrop ND-1000. The integrity of RNA was assessed by electrophoresis on a denaturing agarose gel. The sample preparation and microarray hybridization were performed based on Arraystar’s in-house protocols (Rockville, MD).

Briefly, total RNAs were digested with Rnase R (Epicentre, Inc.) to remove linear RNAs and enrich circular RNAs. Then, the enriched circular RNAs were amplified and transcribed into fluorescent cRNA utilizing a random priming method (Arraystar Super RNA Labeling Kit; Arraystar). The labeled cRNAs were purified by RNeasy Mini Kit (Qiagen) and hybridized onto the Arraystar Mouse circRNA Array V2(8 × 15 K, Arraystar). The concentration and specific activity of the labeled cRNAs (pmol Cy3/μg cRNA) were measured by NanoDrop ND-1000. 1 μg of each labeled cRNA was fragmented by adding 5 μl 10 × Blocking Agent and 1 μl of 25 × Fragmentation Buffer, then heated at 60 °C for 30 min, finally 25 μl 2 × Hybridization buffer was added to dilute the labeled cRNA. 50 μl of hybridization solution was dispensed into the gasket slide and assembled to the circRNA expression microarray slide. The slides were incubated for 17 h at 65 °C in an Agilent Hybridization Oven. The hybridized arrays were washed, fixed and scanned using the Agilent Scanner G2505C (Protocol adapted from in-house protocols developed by Arraystar (Rockville, MD).

### Hierarchical clustering

Hierarchical clustering of circRNAs in all samples was conducted using euclidean clustering for computing dissimilarity between rows and between columns. The expression levels of circRNAs were represented by a color scale where blue represents low expression levels and red represents high expression levels. Each column represents a unique sample type and each row represents a distinct circRNA (Supplementary Fig. 1).

**Figure 1:**
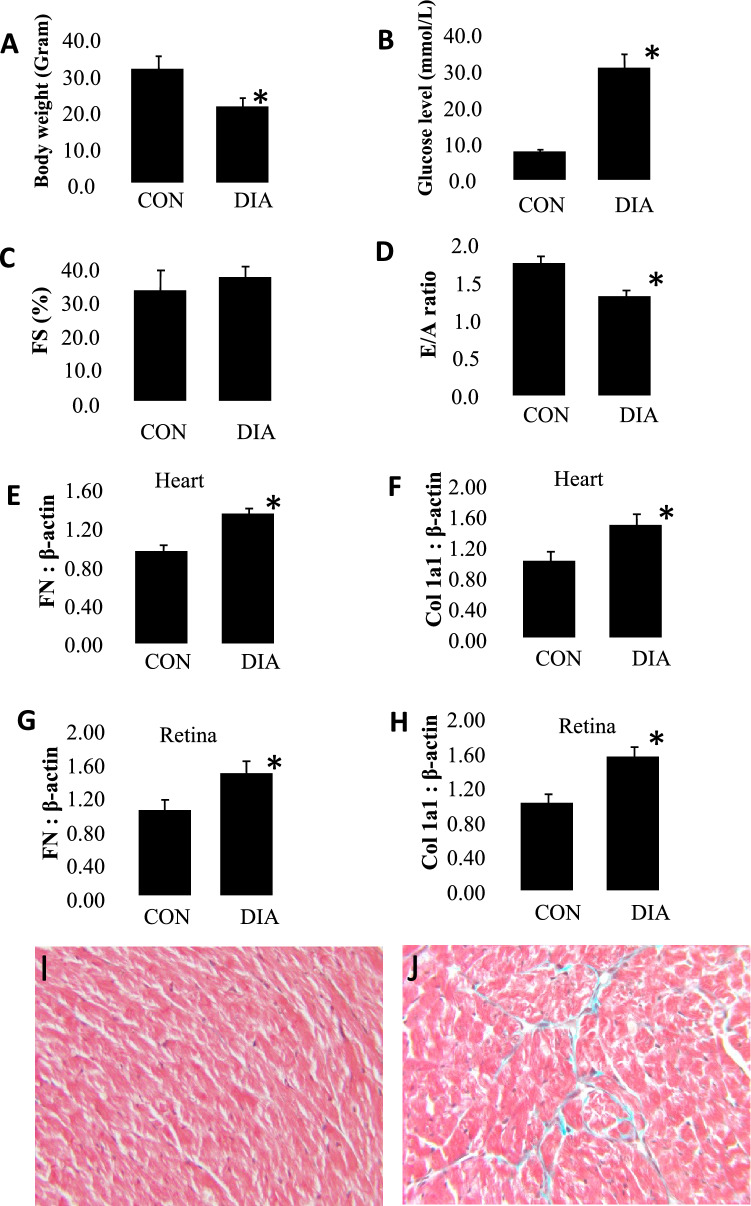
Diabetic (DIA) mice showed animals showed (**A**) reduced body weight and (**B**) hyperglycemia following 2 mo of diabetes compared with non-diabetic age- and sex-matched controls (CON). Echocardiographically diabetic animals also showed (**C**) increased fractional shortening (FS), (**D**) reduced mitral inflow pattern (E/A ratio). Analysis of (**E, F**) cardiac and (**G, H**) retinal tissues showed increased mRNA expression of fibronectin (FN) and collagen 1α1 (Col1a1) (*P = 0.05 or less vs CON, n = 6/group). (**I**) Trichrome stains showed focal scarring and collagen deposition (green stain in the myocardium of the diabetic animals. Such collagen deposition was not seen in the heart of non-diabetic animals. Such collagen deposition was not seen in the heart of (**I**) non-diabetic animals (magnification same for **I** and **J**).

### Differentially expressed circRNAs

Agilent Feature Extraction software (version 11.0.1.1) was used to analyze acquired array images. Quantile normalization and subsequent data processing were performed using the R software limma package^31^. circRNAs that had flags of ‘present’ or ‘marginally present’ for at least 3 out of 12 samples (as defined by GeneSpring software) were retained for further differential analyses. R (version 4.0.4) was used for all downstream data analysis. To mitigate batch effect, harman correction^32^ was implemented with a confidence limit of 0.875. Reducing the confidence limit further was found to cause a loss of biological information. Correction of batch effect was confirmed through analysis of pre-correction and post-correction principal component analysis. A total of eight comparisons were made as described in Table 1. Equal variance two-sided paired t-tests were conducted for the retina vs heart comparisons and equal variance two-sided unpaired t-tests were conducted for the control vs diabetic comparisons. Differentially expressed circRNAs were defined using a p-value threshold of 0.005 and an absolute fold change value threshold of 1.25.

**Table 1:** Overview of the eight contrasts analyzed. H = Heart, R = Retina, C = Control, D = Diabetic.

Tissue comparison (paired t-test)	Diabetic comparison (unpaired t-test)
HC vs RC (upregulated, downregulated)	HC vs HD (upregulated, downregulated)
HD vs RD (upregulated, downregulated)	RC vs RD (upregulated, downregulated)

### GO term & KEGG pathway analysis

For each of the 8 directional comparisons, the 100 most significant genes were inputted into GO term and KEGG pathway analysis. The universe consisted of all unique genes corresponding to circRNA probes on the microarray. The genes corresponding to the differentially expressed circRNAs for each GO term and KEGG pathway were extracted. GO term and KEGG pathway analysis was conducted using *goana* and *kegga* functions in the limma R package.

### circRNA/microRNA interactions

circRNA/microRNA interactions were predicted with Arraystar's home-made miRNA target prediction software based on TargetScan^33^ and miRanda^34^. The top 5 circRNA/microRNA interactions were prioritized by using the miRanda structure score.

### miRNA to circRNA matchup

miRNA 1, miRNA 133a, miRNA-320, miRNA-195, miRNA-200b, miRNA-146a, and miRNA-9 have been previously established as differentially expressed miRNAs in diabetic tissue. We searched the tissue-specific circular RNAs database^35^ for these miRNAs and their associated circRNAs. From these identified circRNA's, we determined the circRNAs that were also differentially expressed as determined by our analysis. Specifically, miRNAs associated with circRNAs from the TSCD database from any tissue other than testis, were compared with our differentially expressed circRNAs. Only complete overlaps were considered valid for this analysis.

## Results

### Diabetic animals showed features of diabetic dysmetabolism

We initially established weather the mice demonstrate features characteristic of diabetic dysmetabolism. Following STZ induction, the mice and age and sex-matched controls were monitored for a period of 2 mo. Hyperglycemia was evident in the diabetic animals along with reduced body weight (Fig. 1) and with polyuria, glycosuria (not shown), distinctive of poorly controlled diabetes. No such changes were seen in the non-diabetic control mice.

### Diabetic animals showed characteristic transcriptional and cardiac functional alterations

We performed functional analysis in the heartandechocardiographic assessment prior to sacrific. We have previously demonstrated that cardiac dysfunction, manifested as abnormalities of cardiac contractility is a characteristic feature of diabetic cardiomyopathy^36^. Hence, we examined whether these animals show similar functional defects. As expected, increased FS and reduced E/A ratio was present in the diabetic mice compared to non-diabetic control mice (Fig. 1).

To confirm whether these animals developed diabetes induced alterations of specific transcripts, we measured extracellular matrix (ECM) protein transcripts. Increased ECM protein production is a characteristic feature of all chronic diabetic complications including those involving the retina and heart^1,4,10,11^. In the current experiments, we also demonstrated increased ECM protein transcript production both in the heart and in the retina of diabetic animals compared to non-diabetic controls (Fig. 1). At the structural level, such changes were reflected in the trichrome stain where increased collagen deposition was noted in the heart of diabetic mice (Fig. 1).

### circRNA differential expression

All of the contrasts between heart and retinal tissue in both diabetic and control mice showed differential expression in a number of circRNAs. Details of all differentially expressed circRNAs can be found in Supplementary Table 1a-h).

### Tissue specific differences in diabetic circRNA expression shows upregulation of synaptic activity in retinal tissue and cardiac contractile pathways in heart tissue

**Figure 2:**
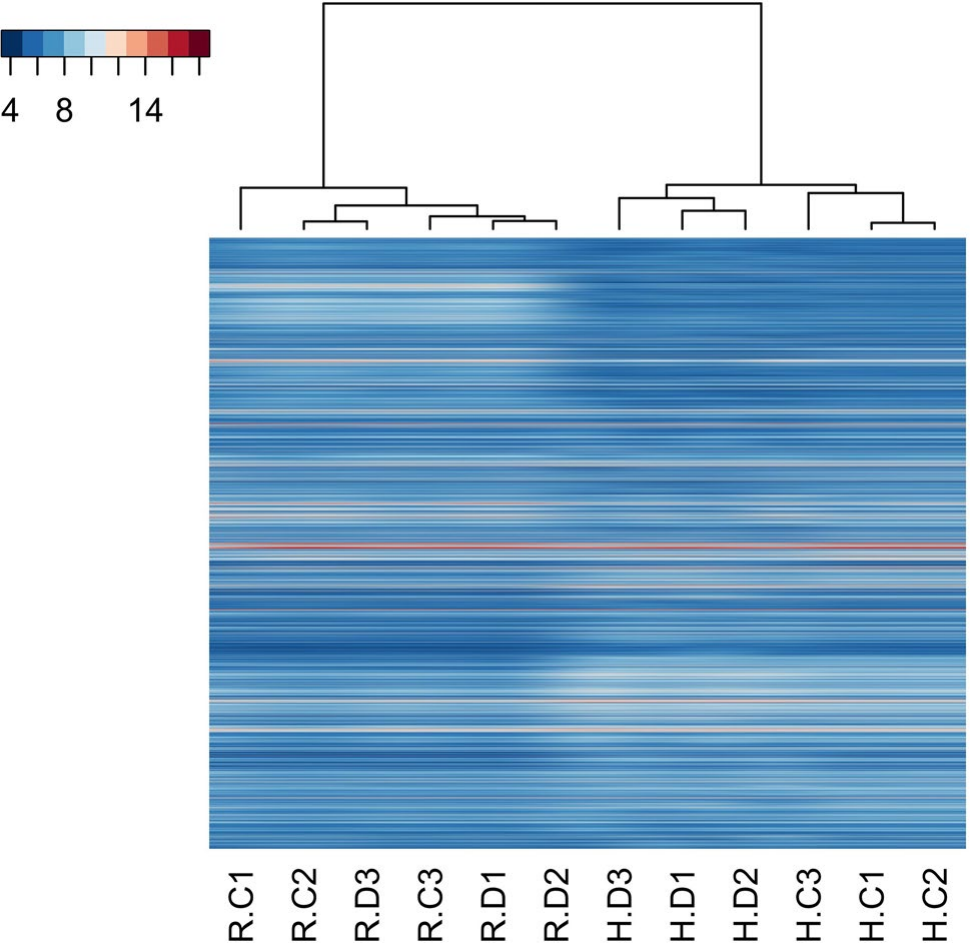
Heatmap hierarchial clustering of all circRNAs across all samples shows global clustering of the retinal (R) and cardiac (H) tissues of diabetic (D) and non-diabetic control (C) mice.

At the basal level there were tissue specific variation of circRNA expression suggesting tissue specific differences of circRNA mediated regulatory mechanisms on gene expression (Fig. 2). In non-diabetic mice, cardiac tissue had significantly upregulated expression of 455 circRNAs (FC > 1.25, p < 5e-3) and retinal tissue had significantly upregulated expression of 236 circRNAs (FC > 1.25, p < 5e-3) (Supplemental Table 1).

The circRNA profiles of cardiac tissue and retinal tissues in mice who were diabetic or non-diabetic showed significant differences. In diabetic mice, cardiac tissue had significantly upregulated expression of 660 circRNAs (FC > 1.25, p < 5e-3) and retinal tissue had significantly upregulated expression of 776 circRNAs (FC > 1.25, p < 5e-3).

**Figure 3:**
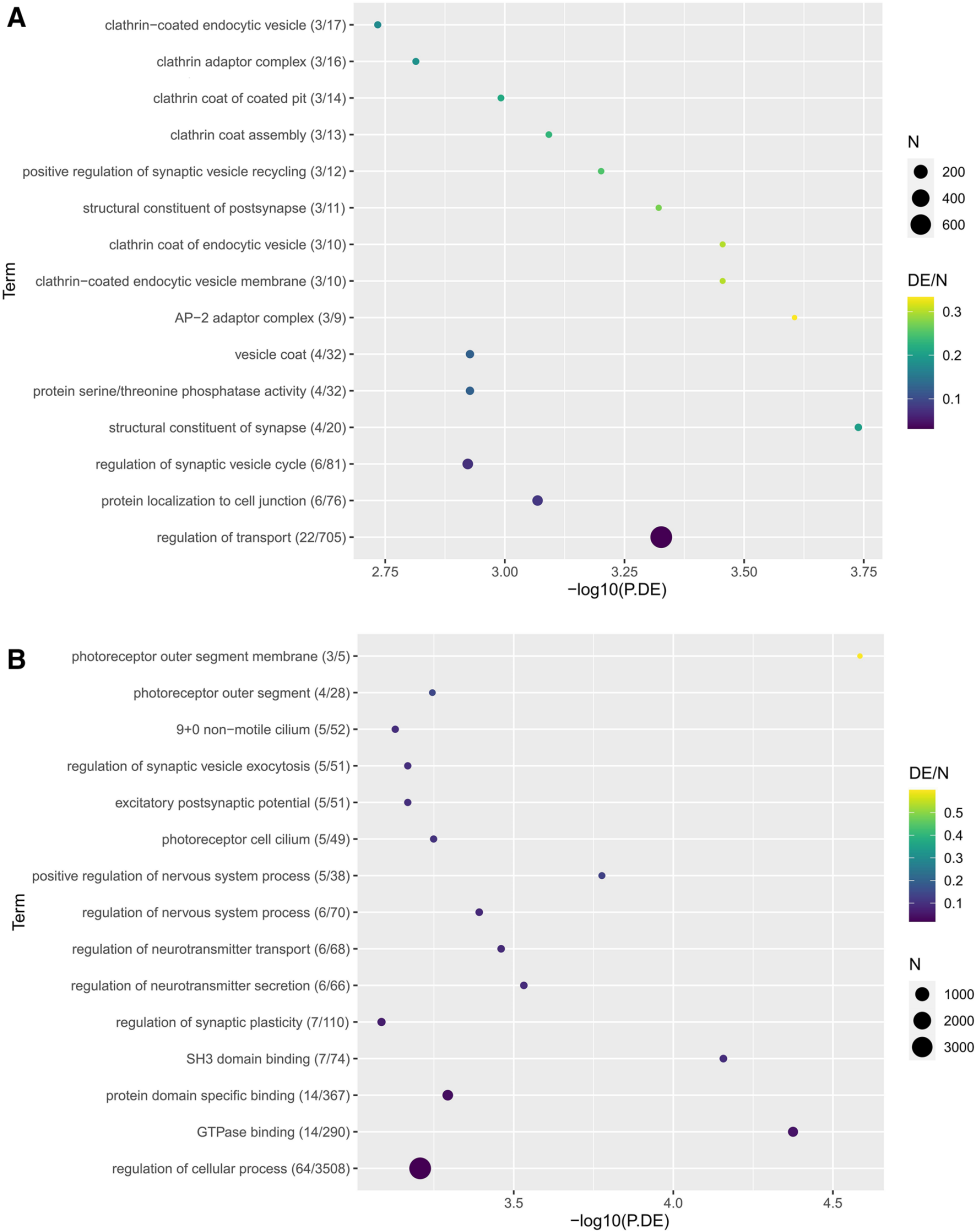

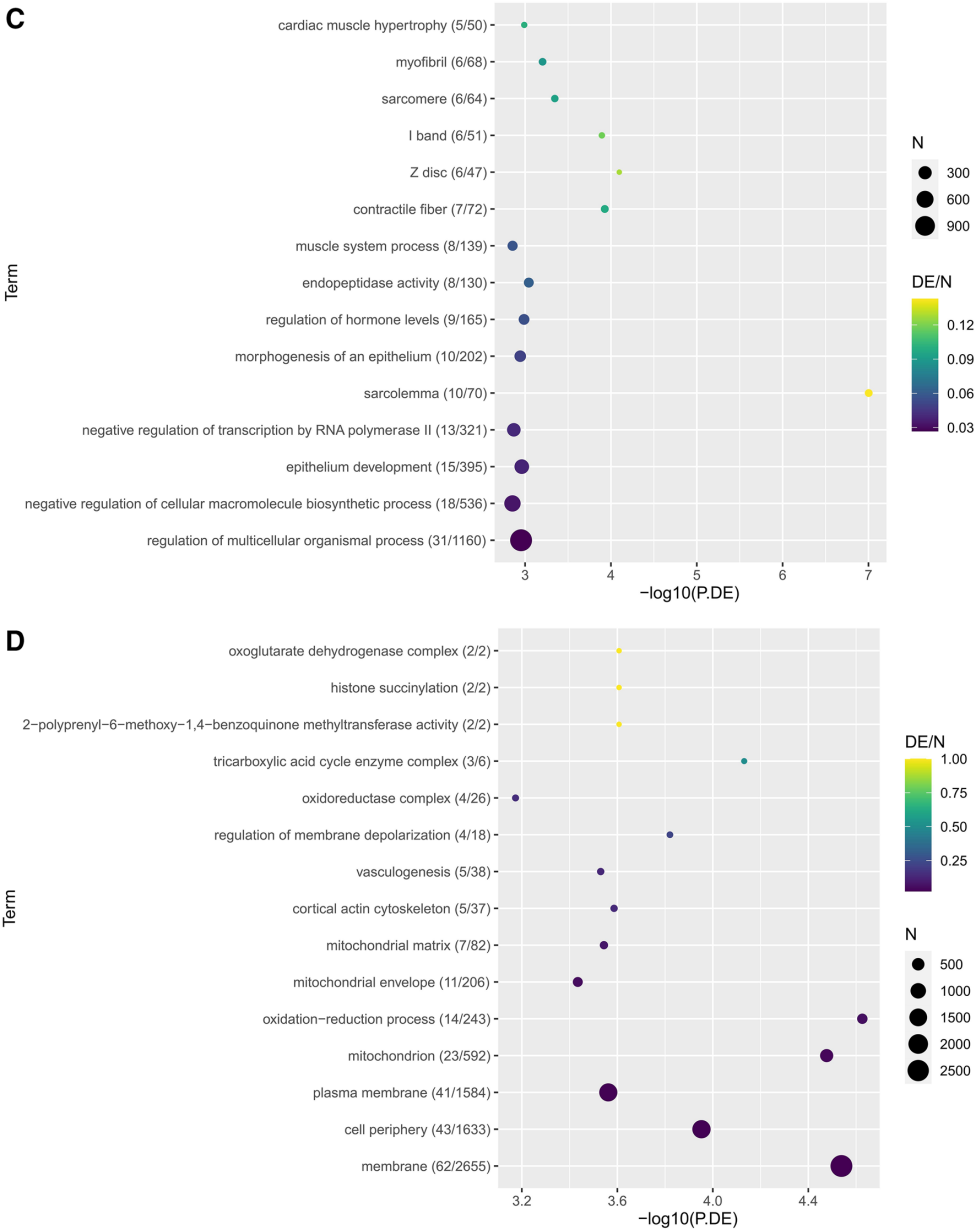
Top 15 (P.DE < 0.05), differentially expressed GO terms by contrast. GO terms are represented with number of DE genes in term/number of genes shown both in brackets after each GO term and controlling dot colour (corresponding heatmap legend). Size of dot represents total N in term. (**A**) HD_RD_down (**B**) HC_RC_down (**C**) HD_RD_up (**D**) HC_RC_up.

KEGG pathway analysis of differential circRNAs suggested pathways relating to synaptic activity were significantly upregulated in diabetic retinal tissue in comparison to diabetic cardiac tissue and included glutamatergic synapse (path:mmu04724, P.DE = 4.49e−4), D-gluatmine and D-glutamate metabolism (pat:mmu00471, P.DE = 3.01e−2) (Table 2). This was supported by the upregulated GO term analysis including structural constituent of synapse (GO:0,098,918, P.DE = 1.83e−4), AP-2 adaptor complex (GO:0,030,122, P.DE = 2.48e−4), and clathrin coat of endocytic vesicle (GO:0,030,128, P.DE = 3.51e−4) (Fig. 3A). Similarly, non-diabetic retinal tissue revealed upregulation in dopaminergic synapse (path:mmu04728, P.DE = 1.32e−2) and spontaneous neurotransmitter secretion (GO:0,061,669, P.DE = 8.92e−05), positive regulation of nervous system process (GO:0,031,646, P.DE = 1.67e−4) and regulation of neurotransmitter secretion (GO:0,046,928, P.DE = 2.94e−4) (Fig. 3B, Table 2).

**Table 2:** Top 15 KEGG pathways by contrast (P < 0.05). H = Heart, R = Retina, C = Control, D = Diabetic.

Contrast	KEGG ID	Pathway	N	DE	P-value
HC_HD Up (HC upregulated)	path:mmu04141	Protein processing in endoplasmic reticulum	70	4	2.35E−02
	path:mmu04973	Carbohydrate digestion and absorption	17	2	2.82E−02
	path:mmu04978	Mineral absorption	18	2	3.14E−02
HC_HD Down (HD upregulated)	path:mmu04020	Calcium signaling pathway	89	6	2.83E−03
	path:mmu00190	Oxidative phosphorylation	26	3	7.85E−03
	path:mmu04260	Cardiac muscle contraction	26	3	7.85E−03
	path:mmu04929	GnRH secretion	33	3	1.52E−02
	path:mmu05415	Diabetic cardiomyopathy	62	4	1.68E−02
	path:mmu04911	Insulin secretion	42	3	2.89E−02
	path:mmu05020	Prion disease	79	4	3.69E−02
	path:mmu05010	Alzheimer disease	117	5	3.85E−02
	path:mmu05022	Pathways of neurodegeneration—multiple diseases	162	6	4.42E−02
	path:mmu04925	Aldosterone synthesis and secretion	51	3	4.73E−02
	path:mmu04713	Circadian entrainment	52	3	4.96E−02
RC_RD Up (RC upregulated)	path:mmu03015	mRNA surveillance pathway	44	4	4.05E−03
	path:mmu04144	Endocytosis	118	6	8.25E−03
	path:mmu04152	AMPK signaling pathway	58	4	1.08E−02
	path:mmu04022	cGMP-PKG signaling pathway	70	4	2.04E−02
	path:mmu03008	Ribosome biogenesis in eukaryotes	40	3	2.16E−02
	path:mmu03022	Basal transcription factors	19	2	3.22E−02
	path:mmu04270	Vascular smooth muscle contraction	49	3	3.65E−02
RC_RD Down (RD upregulated)	path:mmu05143	African trypanosomiasis	6	2	3.20E−03
	path:mmu05146	Amoebiasis	31	3	1.08E−02
	path:mmu04929	GnRH secretion	33	3	1.28E−02
	path:mmu05200	Pathways in cancer	178	7	1.69E−02
	path:mmu04360	Axon guidance	106	5	2.09E−02
	path:mmu04726	Serotonergic synapse	41	3	2.30E−02
	path:mmu04911	Insulin secretion	42	3	2.45E−02
	path:mmu04010	MAPK signaling pathway	115	5	2.86E−02
	path:mmu04550	Signaling pathways regulating pluripotency of stem cells	46	3	3.11E−02
	path:mmu05163	Human cytomegalovirus infection	81	4	3.27E−02
	path:mmu04020	Calcium signaling pathway	89	4	4.39E−02
	path:mmu04725	Cholinergic synapse	55	3	4.88E−02
	path:mmu04935	Growth hormone synthesis, secretion and action	55	3	4.88E−02
	path:mmu04370	VEGF signaling pathway	24	2	4.95E−02
HC_RC Up (HC upregulated)	path:mmu00130	Ubiquinone and other terpenoid-quinone biosynthesis	2	2	2.52E−04
	path:mmu00020	Citrate cycle (TCA cycle)	13	3	1.00E−03
	path:mmu04640	Hematopoietic cell lineage	13	3	1.00E−03
	path:mmu03320	PPAR signaling pathway	21	3	4.25E−03
	path:mmu01100	Metabolic pathways	477	15	8.13E−03
	path:mmu04512	ECM-receptor interaction	28	3	9.67E−03
	path:mmu04810	Regulation of actin cytoskeleton	87	5	1.23E−02
	path:mmu04122	Sulfur relay system	1	1	1.60E−02
	path:mmu01200	Carbon metabolism	37	3	2.07E−02
	path:mmu04971	Gastric acid secretion	37	3	2.07E−02
	path:mmu00564	Glycerophospholipid metabolism	41	3	2.71E−02
	path:mmu00232	Caffeine metabolism	2	1	3.17E−02
	path:mmu01240	Biosynthesis of cofactors	45	3	3.45E−02
	path:mmu00071	Fatty acid degradation	19	2	3.61E−02
	path:mmu04714	Thermogenesis	82	4	4.14E−02
HC_RC Down (RC upregulated)	path:mmu04728	Dopaminergic synapse	65	4	1.32E−02
	path:mmu04911	Insulin secretion	42	3	2.12E−02
	path:mmu05163	Human cytomegalovirus infection	81	4	2.74E−02
	path:mmu00603	Glycosphingolipid biosynthesis—globo and isoglobo series	2	1	2.81E−02
	path:mmu04713	Circadian entrainment	52	3	3.69E−02
	path:mmu04935	Growth hormone synthesis, secretion and action	55	3	4.25E−02
	path:mmu05231	Choline metabolism in cancer	58	3	4.85E−02
HD_RD Up (HD upregulated)	path:mmu04713	Circadian entrainment	52	6	1.51E−04
	path:mmu04020	Calcium signaling pathway	89	7	4.73E−04
	path:mmu04921	Oxytocin signaling pathway	68	6	6.61E−04
	path:mmu05020	Prion disease	79	6	1.46E−03
	path:mmu04927	Cortisol synthesis and secretion	32	4	1.50E−03
	path:mmu05414	Dilated cardiomyopathy	38	4	2.86E−03
	path:mmu04723	Retrograde endocannabinoid signaling	63	5	3.02E−03
	path:mmu04724	Glutamatergic synapse	66	5	3.70E−03
	path:mmu04911	Insulin secretion	42	4	4.14E−03
	path:mmu04261	Adrenergic signaling in cardiomyocytes	70	5	4.77E−03
	path:mmu04975	Fat digestion and absorption	8	2	6.50E−03
	path:mmu04742	Taste transduction	25	3	6.83E−03
	path:mmu04925	Aldosterone synthesis and secretion	51	4	8.29E−03
	path:mmu00760	Nicotinate and nicotinamide metabolism	10	2	1.02E−02
	path:mmu04935	Growth hormone synthesis, secretion and action	55	4	1.08E−02
HD_RD Down (RD upregulated)	path:mmu04724	Glutamatergic synapse	66	6	4.49E−04
	path:mmu00471	D-Glutamine and D-glutamate metabolism	2	1	3.01E−02

Diabetic cardiac tissue revealed upregulation in cardiac contractile metabolic pathways including calcium signaling pathway (path:mmu04020, P.DE = 4.73e−4), oxytocin signaling pathway (path:mmu04921, P.DE = 6.61e−4), and adrenergic signaling in cardiomyocytes (path:mmu04261, P.DE = 4.77e−3) (Table 2). This was supported by the observed upregulation in GO terms including sarcolemma (GO:0,042,383, P.DE = 9.95e−08), Z-disc (GO:0,030,018 P.DE = 8.00e−05), and contractile fiber (GO:0,043,292, P.DE = 1.18e−4) (Fig. 3C). Similarly, in non-diabetic cardiac tissue we observed upregulation in cardiac contractile pathways including ubiquinone and other terpenoid-quinone biosynthesis (path:mmu00130, P.DE = 2.52e−4), citrate cycle (path:mmu00020, P.DE = 1.00e−3), and metabolic pathways (path:mmu0110, P.DE = 8.13e−3) (Table 2). This was supported by the observed upregulation in GO terms including oxidation–reduction process (GO:0,055,114, P.DE = 2.36e−05), mitochondrion (GO:0.0005739, P.DE = 3.33e−05), and tricarboxylic acid cycle enzyme complex (GO:0,045,239, P.DE = 7.39e−05) (Fig. 3D). Interestingly, circadian entrainment (path:mmu04713) was upregulated in diabetic cardiac tissue (P.DE = 1.51e−4) and upregulated significantly in control retinal tissue (P.DE = 3.69e−2).

### Disease specific differences in diabetic circRNA expression shows upregulation of diabetic cardiomyopathy pathways

Non-diabetic cardiac tissue had 105 circRNAs whose expression was significantly upregulated (FC > 1.25, p < 5e−3) and diabetic cardiac tissue had 67 circRNAs whose expression was significantly upregulated (FC > 1.25, p < 5e−3). Non-diabetic retinal tissue had 6 circRNAs whose expression was significantly upregulated (FC > 1.25, p < 5e−3) and diabetic retinal tissue had 3 circRNAs whose expression was significantly upregulated (FC > 1.25, p < 5e−3).

Cardiac tissue in diabetic mice revealed upregulation in diabetic cardiomyopathy and cardiac contractility pathways including calcium signaling pathway (path:mmu04020, P.DE = 2.83e−3), oxidative phosphorylation (path:mmu00190, P.DE = 7.85e−3), cardiac muscle contraction (path:mmu04260, P.DE = 7.85e−3), diabetic cardiomyopathy (path:mmu05415, P.DE = 1.68e−2), and insulin secretion (path:mmu04911, P.DE = 2.89e−3) (Table 2). This was supported by the observed upregulation in GO terms including inner mitochondrial membrane protein complex (GO:0,098,800, P.DE = 1.34e−4), regulation of muscle system process (GO:0,090,257, P.DE = 6.16e−4), and mitochondrial protein complex (GO:0,098,798, P.DE = 3.87e−3) (Supplementary Fig. 1D). Similarly, in diabetic retinal tissue we observed upregulation in calcium signaling pathway (path:mmu04020, P.DE = 4.39e−2) and insulin secretion (path:mmu04911, P.DE = 2.45e−2) pathways (Table 2).

Cardiac tissue in non-diabetic mice revealed upregulation in carbohydrate digestion and absorption (path:mmu04973, P.DE = 2.82e−2) (Table 2). Non-diabetic retinal tissue showed upregulation of three circRNAs which were all associated with the Rmst gene (Table 3).

**Table 3:** Top 10 differentially expressed circRNAs (based on pvalue and FC cutoffs) and top 5 associated miRNAs. H = Heart, R = Retina, C = Control, D = Diabetic.

Contrast	circRNA	Gene	tstat	pvalue	FC	miRNA1	miRNA2	miRNA3	miRNA4	miRNA5
HC_HD Up (HC upregulated)	circRNA_19140	Gsdmcl-ps	14.93	1.17E−04	1.33	miR-1187	miR-7047-5p	miR-504-3p	miR-6975-5p	miR-6931-5p
	circRNA_42653	Adam18	14.33	1.38E−04	2.058	miR-7220-3p	miR-693-3p	miR-139-5p	miR-7077-3p	miR-7048-3p
	circRNA_28983	Pdzrn4	13.82	1.59E−04	1.592	miR-107-5p	miR-205-5p	miR-322-5p	miR-103-1-5p	miR-103-2-5p
	circRNA_31587	Matr3	13.41	1.79E−04	1.276	miR-6405	miR-741-3p	miR-7661-5	miR-215-3p	miR-320-3p
	circRNA_21860	Cdk19	12.95	2.05E−04	2.814	miR-7661-5p	miR-7092-3p	miR-1192	miR-7035-5p	miR-7226-5p
	circRNA_30033	Tmem50b	12.34	2.48E−04	1.481	miR-7013-5p	miR-7062-5p	miR-499-3p	miR-8110	miR-500-3p
	circRNA_19327	Kdm4c	12.32	2.49E−04	1.368	miR-5110	miR-6976-5p	miR-3547-5p	miR-7058-5p	miR-7665-5p
	circRNA_41248	Samd4b	12.14	2.64E−04	1.276	miR-7033-5p	miR-6923-5p	miR-664-5p	miR-6940-5p	miR-7081-5p
	circRNA_40163	Ubn2	12.06	2.71E−04	1.406	miR-1903	miR-6958-5p	miR-7058-5p	miR-6981-5p	miR-7087-5p
	circRNA_35908	Sycp1	12.02	2.75E−04	1.347	miR-692	miR-130a-5p	miR-7234-5p	miR-7214-5p	miR-686
HC_HD Down (HD upregulated)	circRNA_39634	Usp42	−24.75	1.58E−05	1.51	miR-149-5p	miR-7087-3p	miR-7033-5p	miR-665-5p	miR-212-5p
	circRNA_22404	Anks1b	−16.83	7.31E−05	1.802	miR-8100	miR-7027-5p	miR-6946-5p	miR-6999-5p	miR-7665-5p
	circRNA_003203	Uqcrfs1	−13.09	1.97E−04	1.523	miR-6974-3p	miR-7649-3p	miR-136-5p	miR-6908-3p	miR-7060-3p
	circRNA_19122	Naa16	−12.13	2.65E−04	1.445	miR-1894-3p	miR-6976-5p	miR-1187	miR-466e-5p	miR-466a-5p
	circRNA_19311	St6galnac3	−10.49	4.67E−04	1.273	miR-670-3p	miR-107-5p	miR-3089-3p	miR-103-1-5p	miR-103-2-5p
	circRNA_010045	Herc2	−10.34	4.94E−04	1.496	miR-6954-3p	miR-188-3p	miR-3067-5p	miR-7044-3p	miR-7667-5p
	circRNA_44896	Clstn2	−10.28	5.06E−04	1.339	miR-7652-5p	miR-497b	miR-6381	miR-7032-5p	miR-337-3p
	circRNA_27871	Vwa8	−10.17	5.26E−04	1.479	miR-7094b-2-5p	miR-6944-5p	miR-6975-5p	miR-207	miR-3076-3p
	circRNA_008226	Asxl3	−10.15	5.30E−04	1.278	miR-205-5p	miR-7004-3p	miR-106a-3p	miR-1190	miR-6364
	circRNA_26720	Arsb	−9.98	5.66E−04	1.626	miR-7067-5p	miR-7002-3p	miR-135a-5p	miR-7058-3p	miR-7020-5p
RC_RD Up (RC upregulated)	circRNA_32225	Nxf1	19.67	3.94E−05	1.316	miR-7064-5p	miR-7046-5p	miR-1955-3p	miR-6988-5p	miR-6910-3p
	circRNA_19351	Camta1	8.698	9.62E−04	1.425	miR-574-5p	miR-466i-5p	miR-1187	miR-466f	miR-466e-5p
	circRNA_43018	Myo9b	7.007	2.18E−03	1.294	miR-207	miR-673-5p	miR-320-5p	miR-7083-3p	miR-497a-5p
	circRNA_19479	Fgfr2	6.279	3.28E−03	1.288	miR-7012-5p	miR-5110	miR-6981-5p	miR-667-5p	miR-7081-5p
	circRNA_018683	Rn45s	6.03	3.81E−03	1.53	miR-1249-5p	miR-7076-5p	miR-7016-5p	miR-7081-5p	miR-5110
	circRNA_011696	Rn45s	5.756	4.52E−03	1.348	miR-6977-5p	miR-7228-5p	miR-7071-5p	miR-7081-5p	miR-3083-3p
RC_RD Down (RD upregulated)	circRNA_22447	Rmst	−6.345	3.16E−03	2.669	miR-6899-3p	miR-8104	miR-6960-5p	miR-5113	miR-7238-3p
	circRNA_011291	Rmst	−6.042	3.79E−03	2.41	miR-145a-5p	miR-145b	miR-204-3p	miR-3079-3p	miR-760-5p
	circRNA_005214	Rmst	−5.834	4.30E−03	2.777	miR-1187	miR-466c-5p	miR-466a-5p	miR-466e-5p	miR-466p-5p
HC_RC Up (HC upregulated)	circRNA_44683	Mto1	528.7	3.58E−06	1.264	miR-1903	miR-7091-3p	miR-5625-3p	miR-6901-3p	miR-3099-5p
	circRNA_40057	Cald1	330.8	9.14E−06	1.797	miR-6946-3p	miR-320-5p	miR-207	miR-6971-3p	miR-6961-3p
	circRNA_38761	Corin	313.4	1.02E−05	2.872	miR-672-3p	miR-377-3p	miR-7687-3p	miR-5626-5p	miR-668-3p
	circRNA_27408	Sh3bp5	278.7	1.29E−05	2.746	miR-6537-5p	miR-294-5p	miR-511-5p	miR-34a-5p	miR-292b-5p
	circRNA_34706	Dstn	237.3	1.78E−05	1.753	miR-29b-2-5p	miR-7116-3p	miR-29a-5p	miR-3073b-3p	miR-674-5p
	circRNA_26510	Ptdss1	215.4	2.15E−05	1.461	miR-3090-3p	miR-148b-5p	miR-6970-5p	miR-7050-5p	miR-3073a-3p
	circRNA_012938	Mmp15	205.5	2.37E−05	4.091	miR-6998-5p	miR-1231-5p	miR-6339	miR-7062-5p	miR-7672-5p
	circRNA_21676	Utrn	163.3	3.75E−05	2.534	miR-7116-3p	miR-207	miR-1903	miR-141-5p	miR-670-3p
	circRNA_003905	Capns1	142.6	4.92E−05	2.404	miR-540-5p	miR-1982-3p	miR-6913-3p	miR-668-3p	miR-1198-3p
	circRNA_38141	Cd36	140.8	5.05E−05	13.473	miR-205-3p	miR-3475-3p	miR-202-5p	miR-378a-3p	miR-378c
HC_RC Down (RC upregulated)	circRNA_013049	Txndc11	−510.6	3.84E−06	4.156	miR-5110	miR-1960	miR-1898	miR-21a-3p	miR-7012-5p
	circRNA_33958	Zfp385b	−163.7	3.73E−05	3.537	miR-297a-5p	miR-7056-5p	miR-466c-5p	miR-1249-5p	miR-6954-5p
	circRNA_34247	Fmn1	−103.8	9.27E−05	5.855	miR-3099-5p	miR-450a-2-3p	miR-7231-3p	miR-6999-3p	miR-7063-5p
	circRNA_41274	Zfp382	−73.39	1.86E−04	1.741	miR-7214-5p	miR-3090-5p	miR-6905-5p	miR-143-3p	miR-6914-3p
	circRNA_008959	Cpsf6	−72.14	1.92E−04	3.533	miR-7056-5p	miR-6972-5p	miR-6934-5p	miR-6769b-5p	miR-7672-5p
	circRNA_012479	Grik1	−62.42	2.57E−04	8.828	miR-6982-5p	miR-7047-5p	miR-6965-5p	miR-7090-3p	miR-7665-5p
	circRNA_006286	Elf2	−62.09	2.59E−04	6.854	miR-149-5p	miR-7039-3p	miR-7684-5p	miR-7676-5p	miR-6932-3p
	circRNA_30654	Kdm4b	−60.97	2.69E−04	2.564	miR-7686-5p	miR-3081-3p	miR-5132-5p	miR-1946a	miR-6769b-5p
	circRNA_19132	Rims2	−59.26	2.85E−04	49.851	miR-5110	miR-7661-5p	miR-7665-5p	miR-1249-5p	miR-6976-5p
	circRNA_005039	Elf2	−59.24	2.85E−04	12.306	miR-7039-3p	miR-7684-5p	miR-7676-5p	miR-6932-3p	miR-5709-3p
HD_RD Up (HD upregulated)	circRNA_23275	Mgat1	643.1	2.42E−06	2.476	miR-667-5p	miR-6923-5p	miR-149-3p	miR-7052-3p	miR-344d-2-5p
	circRNA_27178	Adk	380.5	6.91E−06	2.931	miR-7215-5p	miR-6929-3p	miR-6933-5p	miR-7668-3p	miR-6340
	circRNA_27753	Ccar2	260.8	1.47E−05	2.421	miR-6919-3p	miR-7089-3p	miR-9768-3p	miR-6961-3p	miR-6971-3p
	circRNA_22083	Lrrc20	237.4	1.77E−05	2.566	miR-7033-5p	miR-323-5p	miR-365–1-5p	miR-1902	miR-7656-3p
	circRNA_43395	Kctd19	171.6	3.39E−05	2.074	miR-6919-3p	miR-6940-5p	miR-29b-2-5p	miR-7033-5p	miR-3473b
	circRNA_20259	Klf7	163.2	3.76E−05	1.517	miR-5110	miR-504-3p	miR-5113	miR-6981-5p	miR-6922-5p
	circRNA_013661	Mllt3	160.8	3.87E−05	2.333	miR-433-3p	miR-6938-5p	miR-1188-5p	miR-7074-5p	miR-421-5p
	circRNA_24171	Thra	158.2	4.00E−05	1.26	miR-383-3p	miR-1982-5p	miR-705	miR-7040-3p	miR-1906
	circRNA_28134	Sepp1	146	4.69E−05	4.621	miR-500-5p	miR-362-5p	miR-3075-3p	miR-6964-3p	miR-1198-5p
	circRNA_004757	Pcsk5	140.6	5.06E−05	1.491	miR-7085-3p	miR-7007-3p	miR-143-5p	miR-6516-5p	miR-7682-3p
HD_RD Down (RD upregulated)	circRNA_011391	Anks1b	−334.8	8.92E−06	27.354	miR-141-5p	miR-7026-5p	miR-22-5p	miR-6899-3p	miR-674-3p
	circRNA_016623	Sntg1	−255.5	1.53E−05	9.674	miR-7674-5p	miR-666-5p	miR-3061-5p	miR-7031-5p	miR-8111
	circRNA_28683	Khdrbs3	−208.1	2.31E−05	16.921	miR-6344	miR-7009-3p	miR-7116-3p	miR-1903	miR-6964-3p
	circRNA_41367	Sergef	−181.1	3.05E−05	6.284	miR-6919-3p	miR-8103	miR-6996-5p	miR-207	miR-298-5p
	circRNA_40528	Gmcl1	−161.4	3.84E−05	1.419	miR-370-3p	miR-302c-3p	miR-466c-5p	miR-6340	miR-7037-3p
	circRNA_25316	Ppm1a	−160.5	3.88E−05	2.847	miR-466o-3p	miR-466m-3p	miR-466i-3p	miR-466q	miR-669c-3p
	circRNA_012479	Grik1	−157.5	4.03E−05	7.988	miR-6982-5p	miR-7047-5p	miR-6965-5p	miR-7090-3p	miR-7665-5p
	circRNA_39953	Ccdc136	−138.2	5.24E−05	2.788	miR-367-5p	miR-7649-3p	miR-1950	miR-320-3p	miR-7030-3p
	circRNA_31233	Mpp7	−136.2	5.39E−05	4.615	miR-670-3p	miR-7649-5p	miR-677-3p	miR-107-5p	miR-130a-5p
	circRNA_40570	9530026P05Rik	−131.9	5.74E−05	1.937	miR-136-5p	miR-6992-5p	miR-29b-2-5p	miR-6965-3p	miR-7230-5p

Given the previous identification by our group of miRNA’s that are known to be associated with these outcomes^4,8,36–38^, we queried miRNA match-ups between those candidates (miR 1, 133a, 320, 195, 200b, 146a and 9) for all differentially expressed circRNAs in this study and identified 30 circRNA-miRNA pairs (Table 4). Of particular interest are mmu_circRNA_36350 and mmu_circRNA_33461 which are upregulated in control heart as compared to diabetic heart and are known to act as sponges for miR-1 and miR-9, respectively.

**Table 4:** circRNAs of interest that are known sponges for miRNAs of interest. H = Heart, R = Retina, C = Control, D = Diabetic.

Contrast	circRNA	P-value	Fold Change	Chr	Start	End	miRNA of Interest
HC_HD_UP (HC upregulated)	mmu_circRNA_36350	3.45E−04	1.33694001	chr3	157,198,423	157,236,542	miR-1
	mmu_circRNA_33461	6.78E−04	1.751186664	chr2	41,185,869	41,511,627	miR-9
HC_RC_DOWN (RC upregulated)	mmu_circRNA_39320	2.28E−03	4.480517808	chr5	122,555,496	122,611,107	miR-320
	mmu_circRNA_38057	3.32E−03	1.790818772	chr5	5,135,318	5,227,258	miR-320
HD_RD_UP (HD upregulated)	mmu_circRNA_42557	8.06E−05	1.298466276	chr8	11,785,712	11,800,868	miR-146a
	mmu_circRNA_42509	1.06E−03	1.898972281	chr8	3,184,950	3,203,034	miR-320
	mmu_circRNA_28144	1.18E−03	3.977908591	chr15	3,457,929	3,551,722	miR-320
	mmu_circRNA_29733	2.70E−03	1.661465223	chr16	43,232,758	43,302,615	miR-200b
	mmu_circRNA_28157	4.83E−03	2.292235014	chr15	4,091,167	4,128,923	miR-320
	mmu_circRNA_28157	9.80E−04	2.86858259	chr15	4,091,167	4,128,923	miR-320
	mmu_circRNA_38523	1.31E−03	1.630137452	chr5	37,185,682	37,229,503	miR-1
	mmu_circRNA_36601	1.46E−03	2.05759112	chr4	32,827,088	32,860,588	miR-320
	mmu_circRNA_36825	2.08E−03	1.971154472	chr4	56,899,025	56,937,979	miR-320
	mmu_circRNA_25929	3.32E−03	1.600834409	chr12	117,575,554	117,658,403	miR-320
	mmu_circRNA_28143	3.58E−03	2.947010709	chr15	3,457,929	3,551,685	miR-320
	mmu_circRNA_29904	3.88E−03	1.301843893	chr16	70,360,857	70,401,849	miR-320
HD_RD_DOWN (RD upregulated)	mmu_circRNA_33461	9.34E−05	3.34537972	chr2	41,185,869	41,511,627	miR-9
	mmu_circRNA_24372	2.47E−04	6.837369789	chr11	108,498,243	108,664,726	miR-320
	mmu_circRNA_24372	2.47E−04	6.837369789	chr11	108,498,243	108,664,726	miR-320
	mmu_circRNA_005357	5.71E−04	3.148708575	chr3	55,853,770	55,891,674	miR-195
	mmu_circRNA_29397	8.57E−04	4.914103566	chr16	19,673,390	19,701,382	miR-320; miR-9
	mmu_circRNA_005132	2.08E−03	2.938842316	chr19	27,900,778	27,982,946	miR-133a
	mmu_circRNA_39316	3.33E−03	2.461979061	chr5	122,540,303	122,569,039	miR-9
	mmu_circRNA_39316	3.33E−03	2.461979061	chr5	122,540,303	122,569,039	miR-320
	mmu_circRNA_39316	3.33E−03	2.461979061	chr5	122,540,303	122,569,039	miR-146a
	mmu_circRNA_41615	3.55E−03	1.506520541	chr7	66,849,706	66,968,914	miR-133a
	mmu_circRNA_40750	4.62E−03	2.307731979	chr6	112,665,277	112,688,038	miR-320; miR-320; miR-146a
	mmu_circRNA_36350	4.93E−03	1.478189657	chr3	157,198,423	157,236,542	miR-1

## Discussion

In this research, we have demonstrated qualitative and quantitative differences in circRNA expression in two tissues affected in chronic diabetic complications. We used a microarray based approach for analysis due to the fact that circular RNA identification requires high junction read counts which traditional RNA sequencing only provides at prohibitive costs. The Arraystar circRNA used in this study provides unambiguous and reliable circular junction-specific array probes of high sensitivity and specificity. Although both retina and heart were affected by diabetes and show some similarities (eg. ECM protein expression), there is significant structural, functional, and biochemical differences. Hence it is expected that such differences in the regulatory mechanisms exists. Further, the results show the presence of disease specific variations in circRNA expression and discordant GO pathway enrichments which are of particular interest. The comparison of control heart and diabetic heart implicates two major processes to be uniquely upregulated in diabetic heart tissue: (1) endothelial cells: positive regulation of blood vessel endothelial cell migration (P = 7.03E−03) and blood vessel endothelial cell migration (P = 4.28E−02); (2) mitochondria: mitochondrial electron transport, ubiqunol to cytochrome c (P = 1.6E−02) and mitochondrial protein complex (P = 3.87E−03). It has been well established that mitochondrial dysfunction is a characteristic abnormality in all chronic diabetic complications including diabetic cardiomyopathy^6,38^. The current data further support this notion and indicate that the critical mediator of such pathogenetic process are regulated by specific circRNAs.

Similarly, in the comparison of control retina to diabetic retina, extracellular matrix is uniquely upregulated in diabetic retinal tissue as evidenced by the enriched GO terms extracellular matrix (P = 1.09E−02), collagen-containing extracellular matrix (P = 1.10E−02). Further, endothelial to mesenchymal transition (EndMT) was uniquely overrepresented (P = 1.47E−02) along with regulation of mesenchymal cell proliferation (P = 3.09E−02), suggesting a possible unique mechanism for mesenchymal cells in diabetic retina. We have previously demonstrated this association in the retina and heart in diabetes. It has been hypothesized that EndMT may be a key mechanism causing tissue damage in all chronic diseases including diabetic cardiomyopathy and retinopathy^30,38,39^. This research further establishes such changes and identifies novel circRNA mediated regulation of such changes. Also, as previously mentioned, increased ECM protein production is a ubiquitous characteristic feature of chronic diabetic complications^4,8,30,38^. Our data indicate that these processes are further regulated by circRNA expression. It is however interesting to note that endMT related transcripts based on the GO term search were only significantly altered in the retina in diabetes in comparison with non-diabetic controls. However, other endothelial-related terms were upregulated in the diabetic heart (as compared to control heart) such as ‘blood vessel endothelial cell migration’ and ‘positive regulation of blood vessel endothelial cell migration’ and ‘regulation of blood vessel endothelial cell migration’. Failure to be picked up by the GO term in the heart may be related to analysis limitations or may reflect true biological differences. Similarly, GO terms related to mitochondrial processes were not upregulated in diabetic retina, however, several related terms are downregulated in the diabetic retina such as, negative regulation of mitochondrial RNA catabolic process, mitochondrial inner membrane peptidase complex, negative regulation of mitochondrial calcium ion concentration, mitochondrial RNA catabolic process, and regulation of mitochondrial RNA catabolic process. Further experiments and analyses of individual circRNA are needed to delineate these findings.

The pathological processes in chronic diabetic complications are indeed complex. Multiple pathogenetic mechanisms play roles in this concert. Epigenetic mechanisms in the form of acetylation, methylation and alterations of non-coding RNA likely all play a part in this symphony. We have previously demonstrated roles of specific lncRNAs and microRNAs in these processes^8,9,36–38^. Of specific relevance to this project, one of the mechanisms through which circRNA works is by sponging specific miRs. We have previously demonstrated alterations of specific miRs in chronic diabetic complications^4,8,36–38^. Hence, we specifically explored whether some of these miRs are regulated by the altered circRNA identified in this study. As predicted we found some of the altered circRNAs indeed regulate miRNAs known to play significant regulatory roles in diabetic cardiomyopathy and retinopathy.

There are few studies performed in chronic diabetic complications which interrogate circRNA expression levels. Interestingly, one of the differentially expressed circRNAs in the comparison of heart and retina in the diabetic mouse, mmu_circ_000203 has been previously identified to be upregulated in diabetic mouse myocardium^40^, and is downregulated in diabetic heart tissue in this study.

Interestingly, there are significant differences compared to other studies^40–42^. Such differences may result from variation in species (human vs rodent), type of diabetes (Type 1 vs Type 2), or duration of diabetes. Our study was also limited due to the inclusion of small number of animals. Although we monitored 6 animals per group we used 3 animals per group for were used for array analyses. Due to resource-related challenges, we had to focus on the male animals from which we were able to obtain high quality RNA. Nevertheless, the current study demonstrates alterations of circRNAs in two target organs of diabetic complications. However, further studies are required to characterize these changes to establish their role and potential clinical utilities.

In summary, we have demonstrated tissue- and diabetes-specific alterations of several circRNAs in the heart and retina. The current study also indicated regulatory and pathogenetic roles of these molecules in the context of diabetic retinopathy and cardiomyopathy. Understanding these novel pathogenetic mechanisms, may in the future, be useful to develop RNA based treatment strategies.

## Supplementary Information

Supplementary Information 1.

Supplementary Information 2.

Supplementary Information 3.

Supplementary Information 4.
