# Supplementary material for: Circular RNA mediated gene regulation in chronic diabetic complications
Source: Sci Rep. 2021 Dec 9;11:23766. doi: 10.1038/s41598-021-02980-y (PMC8660871; doi:10.1038/s41598-021-02980-y)
Supplement: Supplementary file 1 — Supplementary Information 1. [file 41598_2021_2980_MOESM1_ESM.pdf]

Supplemental Figure 1. Top 15 (P.DE<0.05), differentially expressed GO terms by contrast. GO terms are represented with number of DE genes in term/number of genes shown both in brackets after each GO term and controlling dot colour (corresponding heatmap legend). Size of dot represents total N in term. A) RC_RD_up B) RC_RD_down C) HC_HD_up D) HC_HD_down.

A.

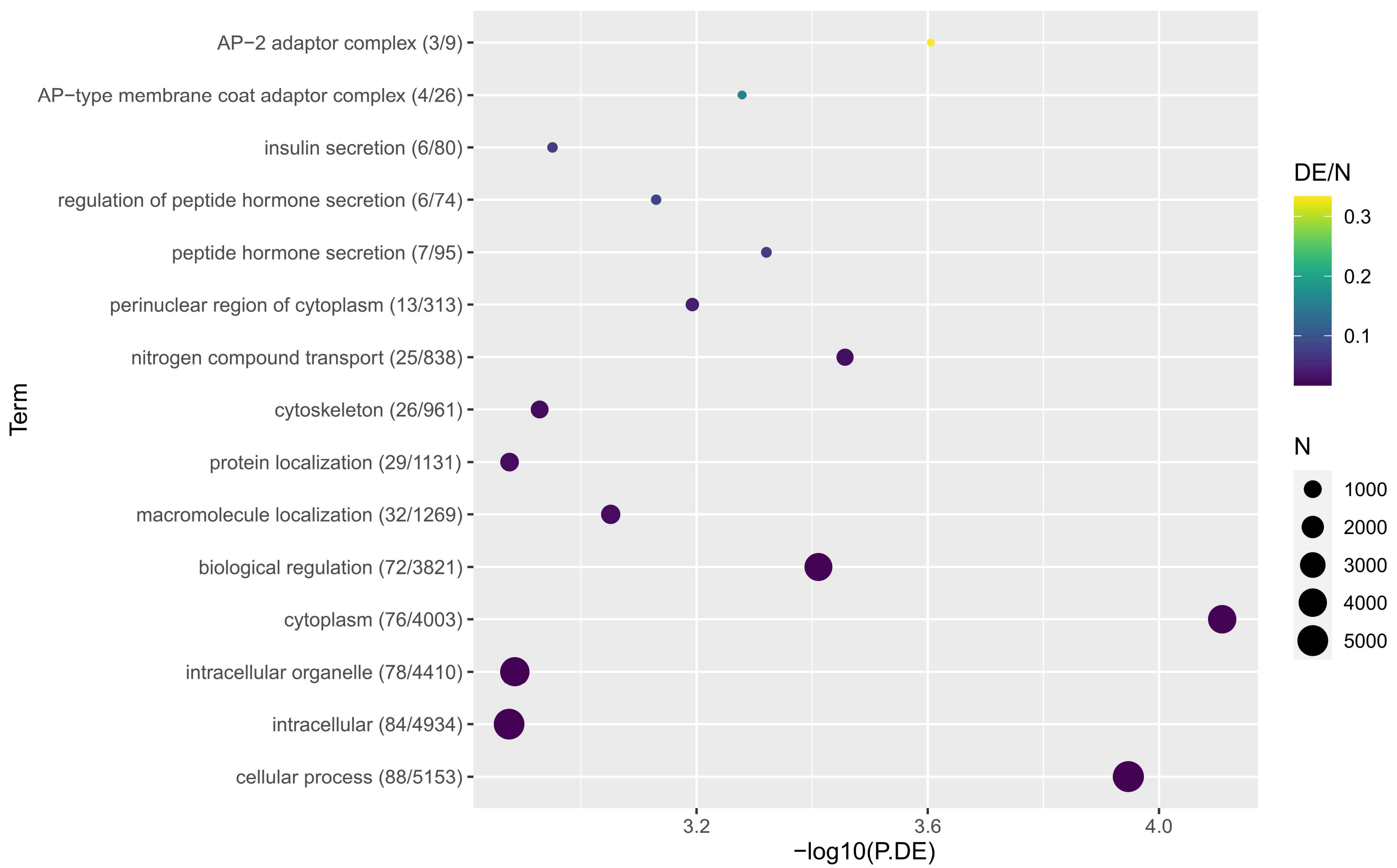

B.

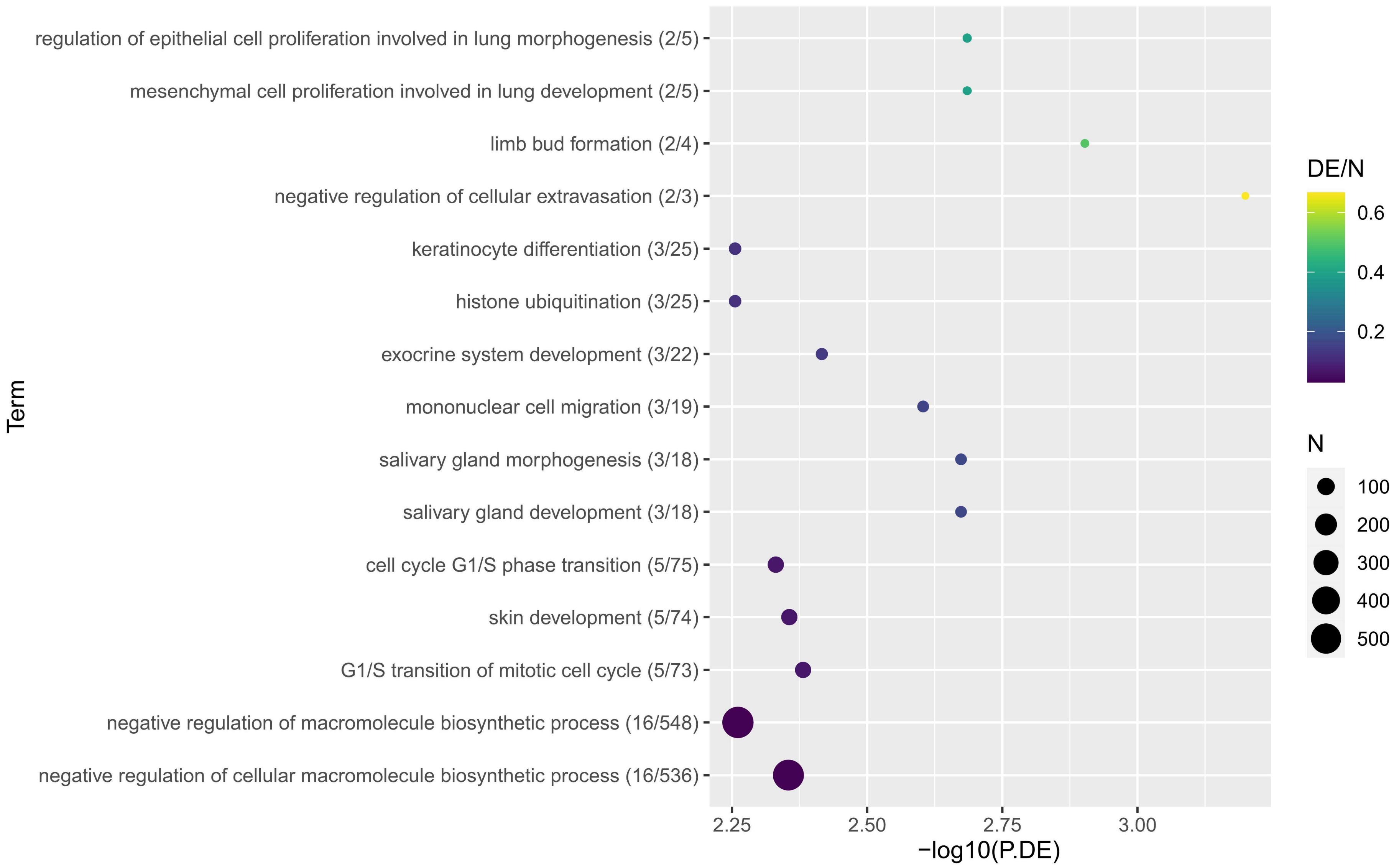

regulation of epithelial cell proliferation involved in lung morphogenesis (2/5)
mesenchymal cell proliferation involved in lung development (2/5)
limb bud formation (2/4)
negative regulation of cellular extravasation (2/3)
keratinocyte differentiation (3/25)
histone ubiquitination (3/25)
exocrine system development (3/22)
mononuclear cell migration (3/19)
salivary gland morphogenesis (3/18)
salivary gland development (3/18)
cell cycle G1/S phase transition (5/75)
skin development (5/74)
G1/S transition of mitotic cell cycle (5/73)
negative regulation of macromolecule biosynthetic process (16/548)
negative regulation of cellular macromolecule biosynthetic process (16/536)
Term
2.25
2.50
2.75
3.00
−log10(P.DE)
DE/N
0.6
0.4
0.2
N
100
200
300
400
500

C.

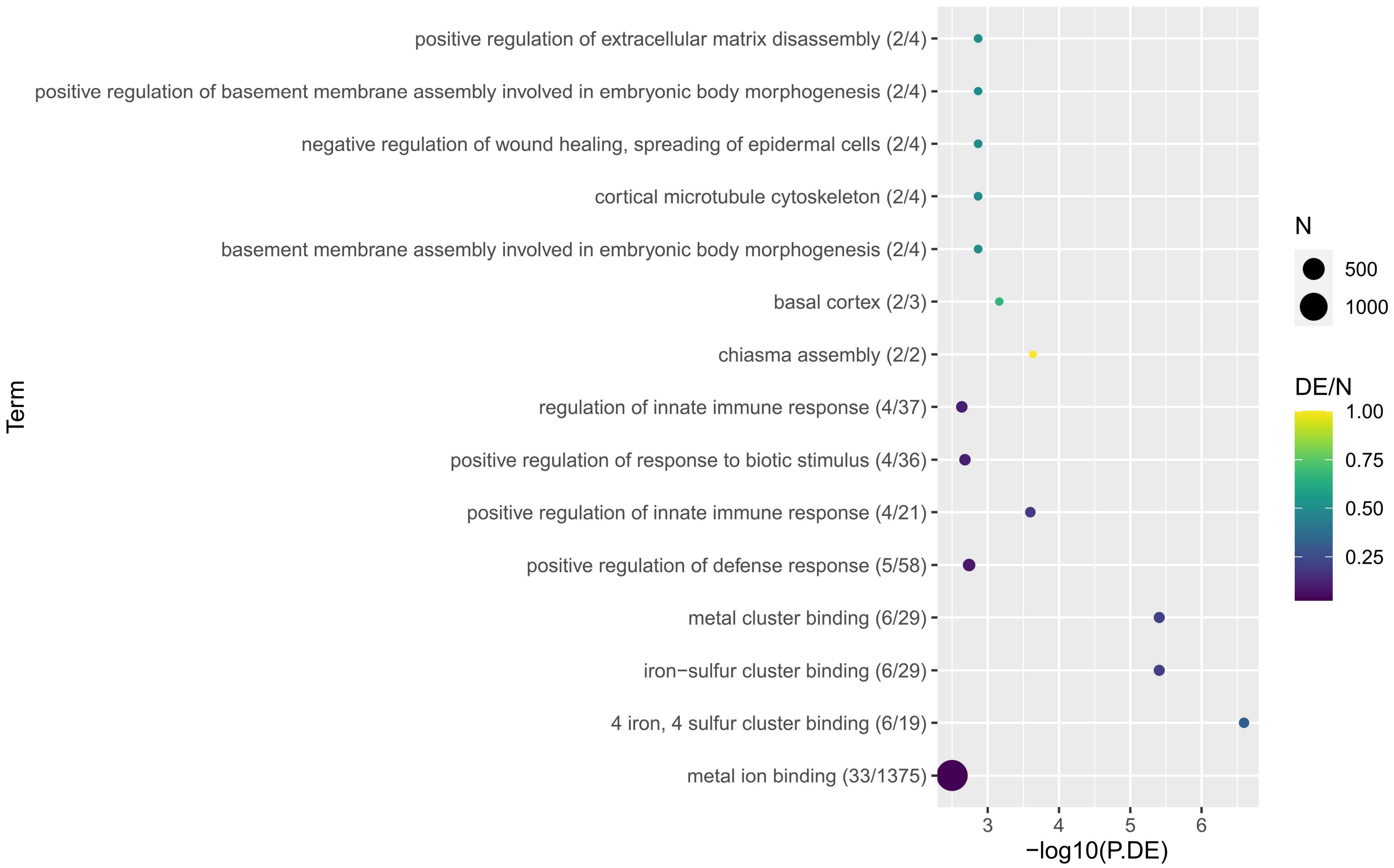
positive regulation of extracellular matrix disassembly (2/4)
positive regulation of basement membrane assembly involved in embryonic body morphogenesis (2/4)
negative regulation of wound healing, spreading of epidermal cells (2/4)
cortical microtubule cytoskeleton (2/4)
basement membrane assembly involved in embryonic body morphogenesis (2/4)
basal cortex (2/3)
chiasma assembly (2/2)
regulation of innate immune response (4/37)
positive regulation of response to biotic stimulus (4/36)
positive regulation of innate immune response (4/21)
positive regulation of defense response (5/58)
metal cluster binding (6/29)
iron−sulfur cluster binding (6/29)
4 iron, 4 sulfur cluster binding (6/19)
metal ion binding (33/1375)
Term
3
4
5
6
−log10(P.DE)
N
500
1000
DE/N
1.00
0.75
0.50
0.25

D.

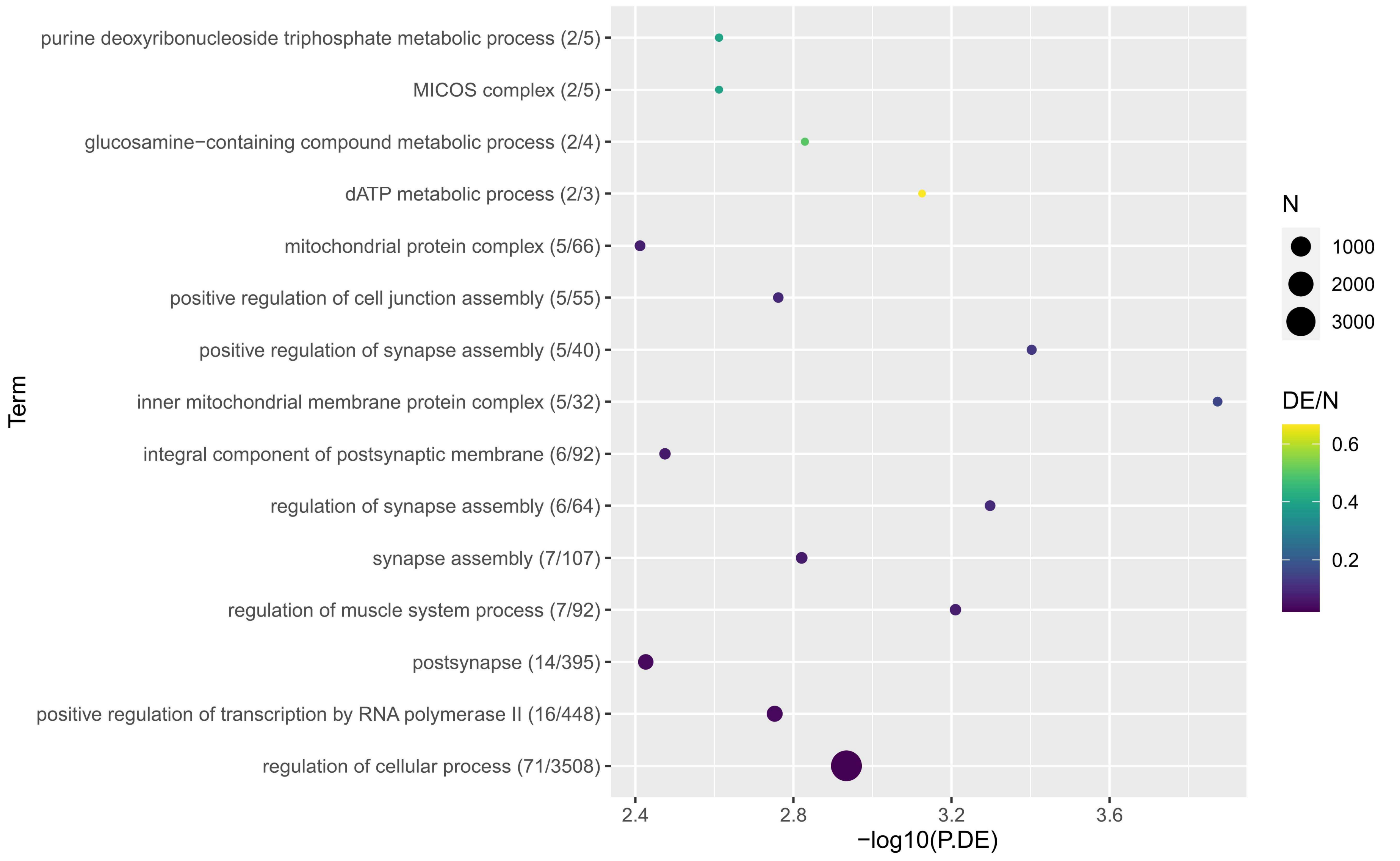

purine deoxyribonucleoside triphosphate metabolic process (2/5)
MICOS complex (2/5)
glucosamine−containing compound metabolic process (2/4)
dATP metabolic process (2/3)
mitochondrial protein complex (5/66)
positive regulation of cell junction assembly (5/55)
positive regulation of synapse assembly (5/40)
inner mitochondrial membrane protein complex (5/32)
integral component of postsynaptic membrane (6/92)
regulation of synapse assembly (6/64)
synapse assembly (7/107)
regulation of muscle system process (7/92)
postsynapse (14/395)
positive regulation of transcription by RNA polymerase II (16/448)
regulation of cellular process (71/3508)
Term
2.4
2.8
3.2
3.6
−log10(P.DE)
N
1000
2000
3000
DE/N
0.6
0.4
0.2
